# Decoy Wnt receptor (sLRP6E1E2)-expressing adenovirus induces anti-fibrotic effect via inhibition of Wnt and TGF-β signaling

**DOI:** 10.1038/s41598-017-14893-w

**Published:** 2017-11-08

**Authors:** Won Jai Lee, Jung-Sun Lee, Hyo Min Ahn, Youjin Na, Chae Eun Yang, Ju Hee Lee, JinWoo Hong, Chae-Ok Yun

**Affiliations:** 1 Institute for Human Tissue Restoration, Department of Plastic & Reconstructive Surgery, Yonsei University College of Medicine, Seoul, Korea; 2 Department of Bioengineering, College of Engineering, Hanyang University, 222 Wangsimni-ro, Seongdong-gu, Seoul, 04763 Korea; 3 Department of Dermatology, Yonsei University College of Medicine, Seoul, Korea

## Abstract

Aberrant activation of the canonical Wingless type (Wnt) signaling pathway plays a key role in the development of hypertrophic scars and keloids, and this aberrant activation of Wnt pathway can be a potential target for the development of novel anti-fibrotic agents. In this study, we evaluated the anti-fibrotic potential of a soluble Wnt decoy receptor (sLRP6E1E2)-expressing non-replicating adenovirus (Ad; dE1-k35/sLRP6E1E2) on human dermal fibroblasts (HDFs), keloid fibroblasts (KFs), and keloid tissue explants. Higher Wnt3a and β-catenin expression was observed in the keloid region compared to the adjacent normal tissues. The activity of β-catenin and mRNA expression of type-I and -III collagen were significantly decreased following treatment with dE1-k35/sLRP6E1E2 in HDFs and KFs. The expression of LRP6, β-catenin, phosphorylated glycogen synthase kinase 3 beta, Smad 2/3 complex, and TGF-β1 were decreased in Wnt3a- or TGF-β1-activated HDFs, following administration of dE1-k35/sLRP6E1E2. Moreover, dE1-k35/sLRP6E1E2 markedly inhibited nuclear translocation of both β-catenin and Smad 2/3 complex. The expression levels of type-I and -III collagen, fibronectin, and elastin were also significantly reduced in keloid tissue explants after treatment with dE1-k35/sLRP6E1E2. These results indicate that Wnt decoy receptor-expressing Ad can degrade extracellular matrix in HDFs, KFs, and primary keloid tissue explants, and thus it may be beneficial for treatment of keloids.

## Introduction

Keloids are skin abnormalities that are unique to humans and characterized by excessive deposition of collagen in the dermis and subcutaneous tissues due to traumatic or surgical injuries^1^. Various mechanisms have been proposed to explain keloid pathogenesis^1–4^. However, treatment of keloids is extremely difficult as keloids frequently form at the site of injury, recur after excision, and always overgrow beyond the boundaries of original wound. Many conventional treatments for keloids have been unsuccessful^5,6^ and thus we explored a novel therapeutic adenovirus (Ad) which can inhibit activities of key pathophysiological pathways of keloid formation.

The canonical Wingless protein (Wnt) signaling profoundly affects developmental processes during embryogenesis, but has an important role for tissue homeostasis in adults^7^. There is considerable evidence that Wnt signaling and its effector β-catenin functions during fibro-proliferative processes characterized by excessive proliferation of mesenchyme cells, which can form hypertrophic scars or neoplasms^8–11^. Wnt proteins are secreted ligands that transmit their signal across the plasma membrane by interacting with Frizzled receptors and low-density lipoprotein receptor-related protein co-receptors (LRP5/6)^12,13^. Receptor-bound Wnt proteins recruit disheveled, axin, adenomatosis polyposis coli (APC), and glycogen synthase kinase (GSK)-3β to the plasma membrane, resulting in stabilization of β-catenin. β-catenin accumulates and translocate to the nucleus, where it interacts with T-cell factor/lymphoid enhancer-binding factor (TCF/LEF) to induce the transcription of Wnt target genes^7,10,12,13^.

Recently, it has been demonstrated that canonical Wnt signaling is necessary for transforming growth factor (TGF)-β1-mediated fibrosis^7,8,14^. Signaling cross-talk between the Wnt/β-catenin, TGF-β1, and Smads signaling is well-documented, where Wnt/β-catenin signaling activates TGF-β1^9,15^ and TGF-β1 promotes Wnt/β-catenin signaling^8,9,15^. TGF-β1 also stimulates canonical Wnt signaling by decreasing the expression of the Wnt antagonist Dickkopf protein-1^7^. Therefore, inhibition of the canonical Wnt pathway can be an effective approach to target TGF-β1 signaling which both can synergistically downregulate fibrogenesis.

LRP6, a member of the LRP superfamily, is required to respond to physiological activation of the canonical Wnt signaling pathway, which leads to the stabilization and nuclear translocation of β-catenin that is essential for fibrogenesis^16^. LRP6 consists of four distinct YWTD β-propeller/EGF-like domain pairs; the first and second YWTD domains (E1 and E2) are required for binding to Wnt^17–19^. Previously, we have demonstrated that soluble Wnt decoy receptor of LRP6 (sLRP6E1E2)-expressing Ad (dE1-k35/sLRP6E1E2), which expresses LRP6’ E1 and E2 domain, prevents Wnt-mediated stabilization of cytoplasmic β-catenin and decreases Wnt/β-catenin signaling^20^.

In this study, we examined the therapeutic potential of dE1-k35/sLRP6E1E2 for treatment of pathologic fibrosis such as keloid. We evaluated the effects of the dE1-k35/sLRP6E1E2 on the expression levels of type-I and -III collagen on human dermal fibroblasts (HDFs), keloid fibroblasts (KFs), and its effect on nuclear translocation of β-catenin and Smad 2/3 complex in HDFs. Additionally, the expression levels of type-I and -III collagen, fibronectin, and elastin in keloid tissue explants transduced with the sLRP6E1E2-expressing Ad were investigated by immunohistochemistry.

## Results

### Wnt3a and β-catenin are upregulated in keloid tissues

As shown in Supplementary Figure S1, both Wnt3a and β-catenin expression levels from keloid tissues were markedly higher by 3.9- and 2.9-fold, respectively, in comparison to extra-lesional normal tissue. As previously reported, these data suggest that Wnt3a and its effector β-catenin protein are actively expressed in keloid tissues^8,21,22^.

### Decoy Wnt receptor sLRP6E1E2 decreases β-catenin/TCF transcriptional activity in HDFs and KFs

**Figure 1:**
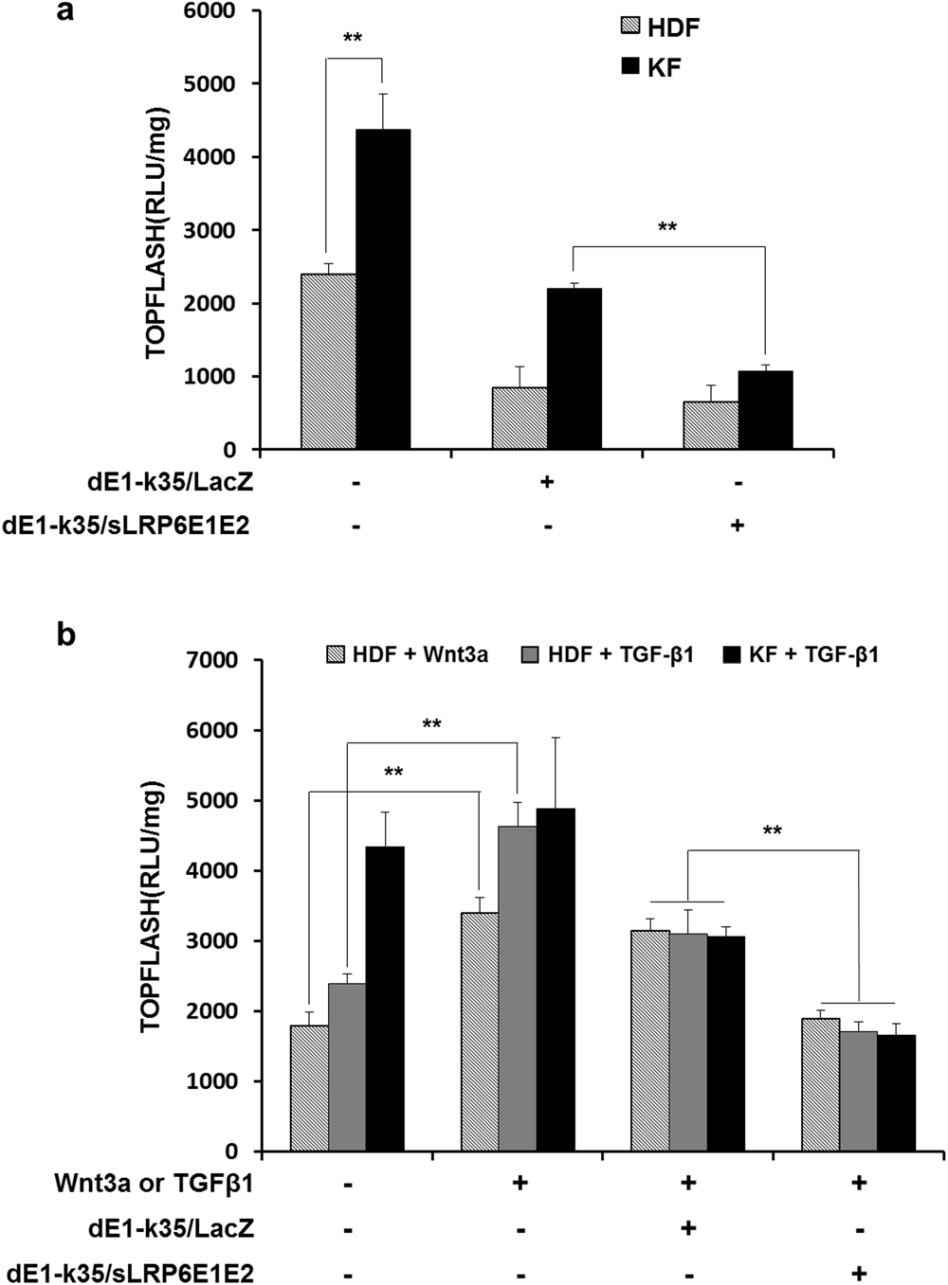
Reduced β-catenin/TCF transcriptional activity by decoy Wnt receptor sLRP6E1E2. TCF/LEF luciferase reporter assay was performed in human dermal fibroblasts (HDFs) and keloid fibroblasts (KFs) to characterize the sLRP6E1E2 effects on the Wnt3a/β-catenin signaling. HDFs and KFs were initially transfected with TOPFLASH (containing wild-type TCF binding sites) plasmid, and then transduced with dE1-k35/LacZ or dE1-k35/sLRP6E1E2 (50 MOI) with canonical Wingless type (Wnt)3a (100 ng/mL) and transforming growth factor (TGF)-β1 (20 ng/mL). Transduction with dE1-k35/sLRP6E1E2 reduced β-catenin/TCF transcriptional activity to a basal level, indicating that sLRP6E1E2 can inhibit the transcription of Wnt target genes. The data are representatives of three independent experiments (**p < 0.01).

To evaluate the ability of sLRP6E1E2 secreted from dE1-k35/sLRP6E1E2 to inhibit Wnt3a/β-catenin signaling, we used a luciferase reporter system activated by β-catenin or TCF. When canonical Wnt signaling is activated, β-catenin will translocate to the nucleus in order to associate with β-catenin/TCF transcription factors which activates transcription of Wnt target genes. Luciferase expression in KF was 1.8-fold higher than those observed in HDFs. dE1-k35/sLRP6E1E2 reduced luciferase expression by 51% compared to the treatment with dE1-k35/LacZ in KFs (Fig. 1a). After treatment with Wnt3a (100 ng/mL) or TGF-β1 (10 ng/mL), luciferase expression level in HDFs was increased by 1.9- or 1.6-fold, respectively, in comparison to untreated HDFs, showing that Wnt3a and TGF-β1 can effectively activate β-catenin/TCF signaling (Fig. 1b). Importantly, luciferase expression was significantly reduced by the treatment with dE1-k35/sLRP6E1E2 compared to the treatment with dE1-k35/LacZ in Wnt3a-activated HDFs, TGF-β1-activated HDFs, and TGF-β1-activated KFs (37%, 45%, and 46%, respectively).

### Decoy Wnt receptor sLRP6E1E2 downregulates mRNA expression of type-I and -III collagen in HDFs and KFs

**Figure 2:**
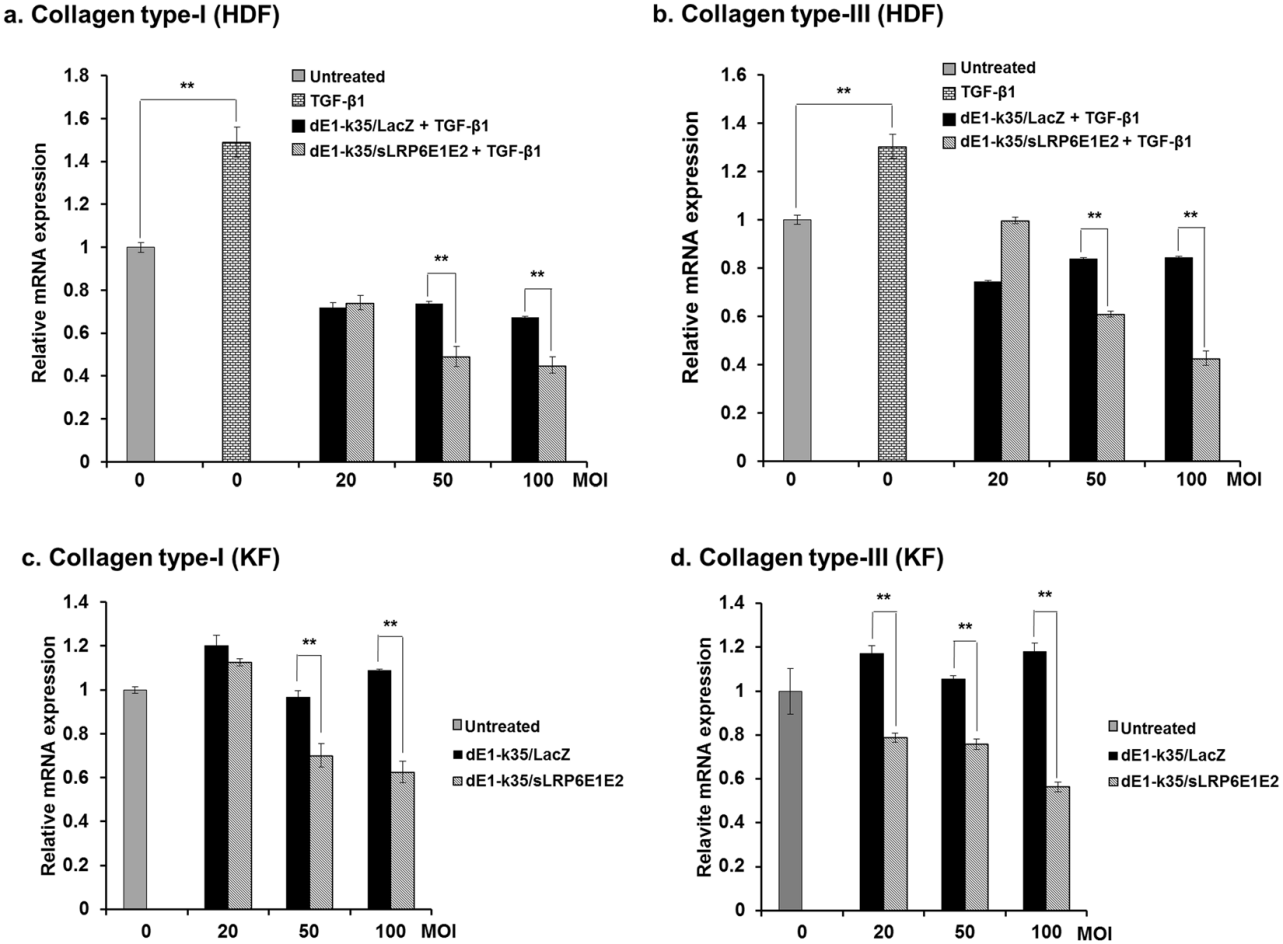
Effect of sLRP6E1E2-expressing adenovirus (Ad) on the mRNA levels of type-I and -III collagen. Quantitative mRNA levels of type-I and -III collagen in HDFs and KFs were assessed by qRT-PCR after transduction with dE1-k35/LacZ or dE1-k35/sLRP6E1E2 (20, 50, and 100 MOI) in the presence of TGF-β1 (20 ng/mL). Both type-I (**a**) and type-III (**b**) collagen mRNA levels in HDFs treated with dE1-k35/sLRP6E1E2 were significantly lower than those in dE1-k35/LacZ-treated HDFs (**p < 0.01). Also, in KFs, type-I (**c**) and type-III (**d**) collagen mRNA levels treated with various amouns of dE1-k35/sLRP6E1E2 were significantly lower than those in dE1-k35/LacZ-treated KFs (**p < 0.01). Data are expressed as mean ± SD of a representative experiment from 3 independent experiments performed with triplicate samples.

To evaluate the potential effect of sLRP6E1E2 on the downregulation of extracellular matrix (ECM) production by inhibition of Wnt3a/β-catenin signaling in HDFs and KFs, the change of type-I and -III collagen mRNA expression levels of HDFs and KFs after treatment with dE1-k35/sLRP6E1E2 or dEl-k35/LacZ were examined. After addition of TGF-β1 (20 ng/mL), type-I and –III collagen mRNA levels were significantly increased in HDFs. However, increased type-I (a) and type-III (b) collagen mRNA levels were significantly attenuated by dE1-k35/sLRP6E1E2 treatment, especially at 50 and 100 multiplicity of infection (MOI)s, when compared to dEl-k35/LacZ-transduced HDFs (Fig. 2a and b). Similar results were obtained in dE1-k35/sLRP6E1E2-transduced KFs (Fig. 2c and d). These results demonstrate that Wnt decoy receptor sLRP6E1E2 can significantly reduce the mRNA levels of type-I and -III collagen, major ECM components, in HDFs and KFs.

### Decoy Wnt receptor sLRP6E1E2 decreases the expression of β-catenin, pGSK-3β, Smad 2/3 complex, and TGF-β1 expression

**Figure 3:**
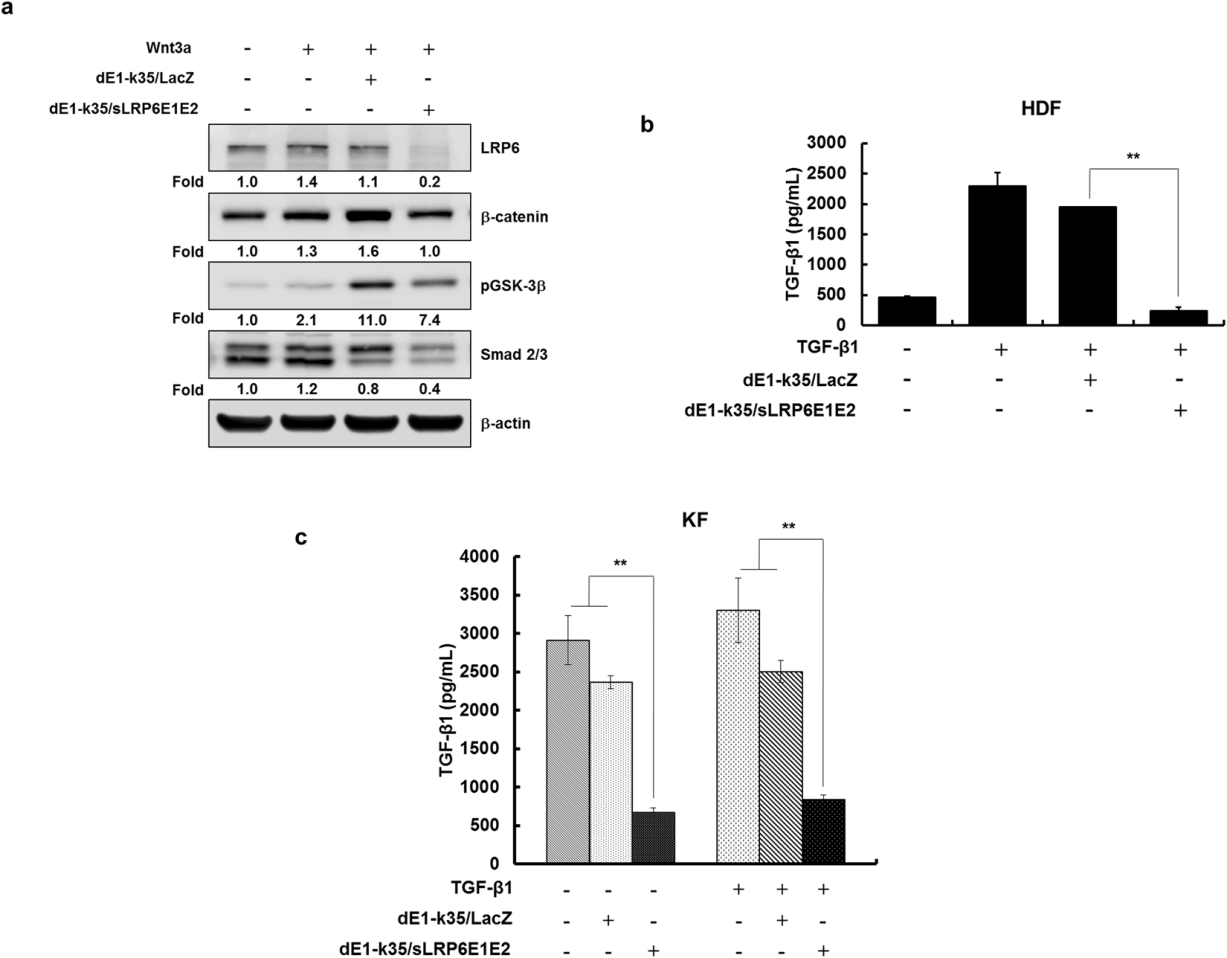
Effect of sLRP6E1E2-expressing Ad on TGF-β1 and Wnt3a/β-catenin signaling pathway. (**a**) Immunoblotting analysis was performed to analyze the expression levels of LRP6, β-catenin, pGSK-3β, and Smad 2/3 complex in HDFs after transduction with either dE1-k35/LacZ or dE1-k35/sLRP6E1E2 (50 MOI) in the presence of Wnt3a. (**b** and **c**) Secreted TGF-β1 protein was measured by ELISA. HDFs and KFs were treated with TGF-β1 to induce fibrosis, and then transduced with dE1-k35/sLRP6E1E2 (50 MOI). Following transduction with dE1-k35/sLRP6E1E2, TGF-β1-activated HDFs (**b**), KFs, and TGF-β1-activated KFs (**c**) exhibited significantly reduced TGF-β1 expression (**p < 0.01). Data are expressed as mean ± SD of a representative expreriment from 3 independent experiments performed with triplicate samples.

We examined the intracellular signaling pathway involved in the anti-fibrotic effect of sLRP6E1E2 on canonical Wnt signaling by analyzing β-catenin, pGSK-3β, and Smad 2/3 complex expression. As shown in Fig. 3a, LRP6 protein level was significantly decreased in Wnt3a-treated HDFs (100 ng/mL) when transduced with dEl-k35/sLRP6E1E2 in comparison to dEl-k35/LacZ. Consequently, β-catenin, pGSK-3β, and Smad 2/3 complex levels were attenuated in dEl-k35/sLRP6E1E2-transduced HDFs than those in dEl-k35/LacZ-transduced HDFs. Further, HDFs and KFs were treated with TGF-β1 to induce fibrosis and subsequently transduced with dE1-k35/sLRP6E1E2 (50 MOI). dEl-k35/sLRP6E1E2-treated HDFs and KFs had significantly lower level of TGF-β1 than dEl-k35/LacZ-treated HDFs and KFs (Fig. 3b and c).

### Decoy Wnt receptor sLRP6E1E2 inhibits translocation of β-catenin and Smad 2/3 to the nucleus

**Figure 4:**
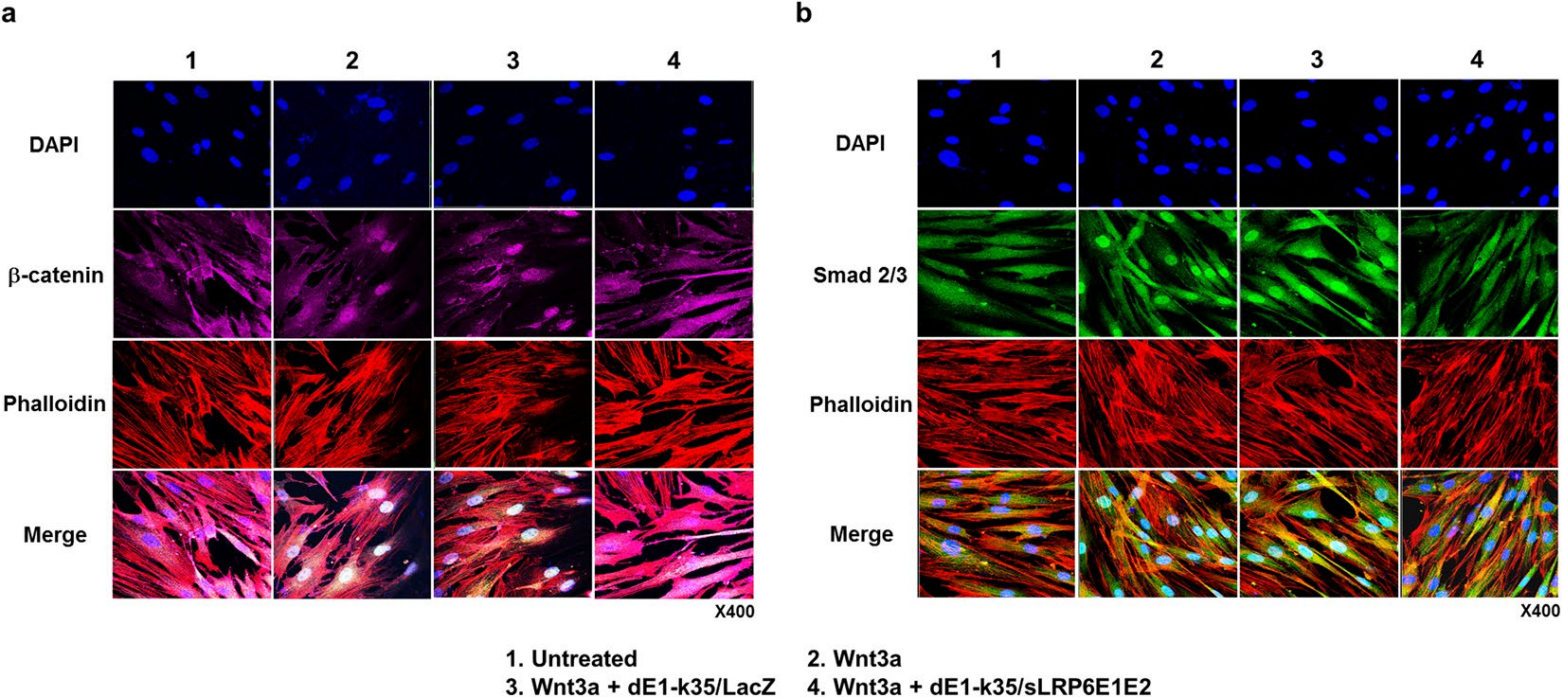
Inhibited nuclear translocation of β-catenin and Smad 2/3 by sLRP6E1E2-expressing Ad. Upon Wnt3a (100 ng/mL) stimulation, the expression of β-catenin and Smad 2/3 was reduced at the cytosols, but increased in the nucleus. In contrast, transduction with dEl-k35/sLRP6E1E2 (50 MOI) decreased nuclear translocation of (**a**) β-catenin and (**b**) Smad 2/3. The data are representatives of three independent experiments.

TGF-β induces heteromeric complexion of Smad 2 and 3 and their concomitant translocation to the nucleus, which is required for efficient TGF-β signal transduction^23^. It has been previously reported that activation of Wnt pathway elicits stabilization and nuclear translocation of β-catenin protein^24^. Nuclear β-catenin accumulation in fibroblastic foci of the patients with systemic sclerosis associated advanced fibrosis has also been reported^25^. Therefore, we examined the extent of nuclear localization of β-catenin and Smad 2/3 by immunofluorescence staining of HDFs after transduction with dE1-k35/LacZ or dE1-k35/sLRP6E1E2 (Fig. 4a and b). In absence of Wnt3a, both β-catenin and Smad 2/3 complex were observed in the cytosols. Upon Wnt3a stimulation, control groups (untreated and dE1-k35/LacZ) showed reduced β-catenin and Smad 2/3 localization at the cytosols, whereas increased Smad 2/3 and β-catenin localization on the nucleus was observed. In contrast, dE1-k35/sLRP6E1E2-transduced cells showed lower levels of nuclear localization of β-catenin and Smad 2/3 than dEl-k35/LacZ-transduced HDFs.

### Decoy Wnt receptor sLRP6E1E2 markedly decreases the expression of Wnt3a, TGF-β1, major ECM components, and MMPs in keloid tissue explants

**Figure 5:**
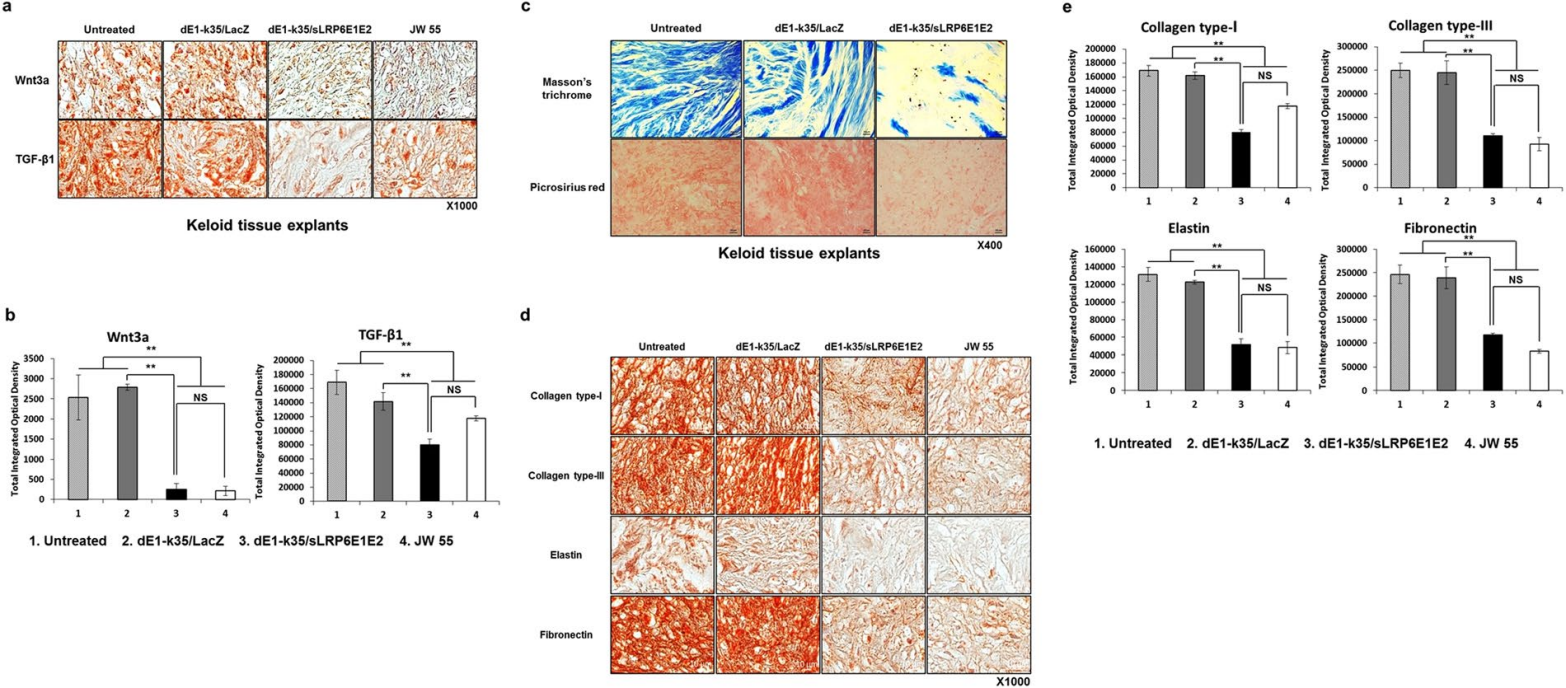
Decreased expression of Wnt3a, TGF-β1, and major ECM components in *keloid tissue explants* by sLRP6E1E2-expressing Ad. (**a**) Immunohistochemical staining demonstrates that the expression levels of both Wnt3a and TGF-β1 are decreased in keloid tissue explants treated with either JW 55 (10 mM) or dE1-k35/sLRP6E1E2 (50 MOI), compared to those in dE1-k35/LacZ-transduced (50 MOI) keloid tissue explants. Original magnification: ×1000 (**b**) Results from immunohistochemical analysis of Wnt3a and TGF-β1 were semi-quantitatively analyzed by MetaMorph^®^ image analysis software (**p < 0.01, *p < 0.05) (**c**) Masson’s trichrome or Picrosirius red staining was performed to assess collagen intensity and deposition in keloid tissue explants. Transduction with dEl-k35/sLRP6E1E2 (50 MOI) markedly decreased collagen intensity and deposition than those transduced with dEl-k35/LacZ (50 MOI). Original magnification: ×400 (**d**) Immunohistochemical staining for type-I and -III collagen, elastin, and fibronectin in keloid tissue explants. Keloid tissue explants treated with JW 55 or dE1-k35/sLRP6E1E2 exhibited markedly attenuated expression level of major ECM components in comparison to dE1-k35/LacZ-transduced keloid tissue explants. Original magnification: ×1000. (**e**) Results from immunohistochemical analysis of major ECM components were semi-quantitatively analyzed by MetaMorph^®^ image analysis software (**p < 0.01). The data are representatives of three independent experiments.

We next evaluated the clinical effect of sLRP6E1E2 in keloid tissue explants through western blot analysis, histological, and immunohistochemical analysis. The decreased levels of Wnt3a and TGF-β1 in keloid tissue explants treated with dEl-k35/sLRP6E1E2 was reduced by 91% and 43%, respectively, in comparison to dE1-k35/LacZ-treated keloid tissue explants (Fig. 5a,b, and Supplementary Figure S2). Although TGF-β1 expression level seems to have decreased in dEl-k35/LacZ-treated samples compared to untreated, there was no statistically significant difference (Fig. 5a and b). Masson’s trichrome and picrosirius red staining revealed excessive deposition of collagen arranged in thick and irregular bundles in keloid tissue explants (Fig. 5c). However, collagen deposition and intensity was markedly decreased in dEl-k35/sLRP6E1E2-treated keloid tissue explants in comparison to those in dEl-k35/LacZ-treated keloid tissue explants. In addition, the expression levels of type-I and -III collagen, elastin, and fibronectin in keloid tissue explants transduced with dEl-k35/sLRP6E1E2 was reduced by 50%, 55%, 58%, and 51%, respectively, in comparison to dE1-k35/LacZ-treated keloid tissue explants. Similar results were obtained when keloids were treated with commercially available Wnt inhibitor JW55 as those observed in dE1-k35/sLRP6E1E2-treated samples, demonstrating that Wnt inhibition can lead to degradation of aberrant keloid ECM (Fig. 5a,b,d, and e). A decrease of type-I and -III collagen protein levels in dEl-k35/sLRP6E1E2-treated keloid tissue explants was confirmed by western blot analysis (Supplementary Figure S3). Further, the expression level of matrix metalloproteinase (MMP)-9 in keloid tissue explants transduced with dEl-k35/sLRP6E1E2 was significantly increased in comparison to dE1-k35/LacZ-treated keloid tissue explants (Supplementary Figure S4).

## Discussion

Aberrant synthesis, accumulation, and organization of ECM molecules such as collagens, microfibrillar proteins and proteoglycans, play significant roles in abnormal scar tissue formation. Excessive ECM accumulation resulting from an imbalance between synthesis and degradation of ECM proteins can lead to hypertrophic scar and keloids. Currently, only available keloid treatment is surgical excision which has limited therapeutic efficacy as keloid recurs soon after the excision and often spread beyond its original margin^2,5,6^. Therefore, novel and effective treatment modality which can degrade accumulated and aberrant collagen deposition of keloids is required.

In present study, we confirmed that both Wnt3a and β-catenin proteins are overexpressed in human primary keloid tissues, suggesting that Wnt signaling pathway may be involved in regulation of collagen expression in keloids. Thus, we designed and constructed soluble Wnt decoy receptor-expressing Ad to inhibit Wnt/β-catenin signaling pathway. Our results demonstrate that dE1-k35/sLRP6E1E2 can attenuate β-catenin/TCF signaling activity in HDFs and KFs. Furthermore, dE1-k35/sLRP6E1E2 also decreased the level of Wnt3a/β-catenin signaling factors such as β-catenin, pGSK-3, and Smad 2/3 complex in Wnt3a-treated HDFs, suggesting that Wnt decoy receptor sLRP6E1E2 can inhibit activity of multiple effectors that are essential to Wnt3a/β-catenin signaling pathway.

Wnt signaling and its effector β-catenin are known to be key regulators in wound healing and has a profound effects on fibroblast activation and fibrogenesis^9,26–31^. Recent studies have demonstrated that aberrant canonical Wnt/β-catenin signaling pathway plays a key role in the development of abnormal organ fibrosis such as hepatic, pulmonary, renal, and skin fibrosis^8,9,12,26–35^. Furthermore, pathologically activated canonical Wnt signaling has been implicated in abnormal wound repair which leads to various fibrotic diseases such as keloid and hypertrophic scar^7,8,26^. These reports suggest that inhibitors targeting canonical Wnt signaling may have a benefit in reduction of collagen expression in keloids^30,36^. Wnt signaling can be divided into canonical and non-canonical cascades. In particular, non-canonical Wnt signaling can occur through planar cell polarity pathway^37–39^ and Wnt/calcium pathways^40,41^, whose effects seem to be β-catenin-independent in that there is no apparent stabilization of cytoplasmic β-catenin^40^. The roles of non-canonical Wnt signaling on abnormal skin fibrosis are less well understood. However, recent reports suggest that non-canonical Wnt pathways could be involved in cardiac hypertrophy via phospholipase-C/Ca2^+^ mobilization and disheveled-protein activation of small GTPases^42^. In this regard, non-canonical Wnt signaling might also be active in keloid and anti-fibrogenic effect of dEl-k35/sLRP6E1E2 on this pathway should be examined in future studies.

TGF-β1 is a potent fibrogenic growth factor that plays a major role in keloid pathophysiology^43,44^. In our study, Ad-mediated expression of sLRP6E1E2 reduced expression of major ECM components in fibroblast and keloid tissue explants. Furthermore, dEl-k35/sLRP6E1E2 potently attenuated TGF-β1 protein secretion in HDFs, KFs, TGF-β1-activated KFs, and keloid tissue explants. A recent study demonstrated that canonical Wnt signaling may also activate the TGF-β1 cascade^7,8,14,45^ and vice versa^9,15^ with increased nuclear accumulation of β-catenin and induction of TCF/Lef reporter activity^28^. Altogether, these findings suggest that signaling cross-talk between Wnt3a and TGF-β signaling pathways is integral to fibrosis^9,30^, thus inhibition of canonical Wnt pathway can be an effective approach to target TGF-β1 signaling to downregulate fibrogenesis.

Previous reports report the nuclear accumulation of β-catenin in fibroblast foci of most idiopathic pulmonary fibrosis samples and experimental lung fibrosis^46,47^, indicating that nuclear translocation of β-catenin is critical to the induction of fibrogenesis through Wnt/β-catenin pathway. Our results indicate that dE1-k35/sLRP6E1E2 can reduce activation of canocical Wnt/β-catenin pathway by inhibiting nuclear translocation of β-catenin and Smad 2/3 complex, which are key effectors of Wnt signaling. dEl-k35/sLRP6E1E2-transduced keloid tissue explants had markedly reduced ECM components and MMP-2 and MMP-9. Taken together, we have demonstrated that dE1-k35/sLRP6E1E2 is highly anti-fibrogenic due to the potent inhibition of Wnt3a/β-catenin and TGF-β1/Smad 2/3 signaling pathway. Although these preliminary findings show that Ad-mediated inhibition of Wnt signaling cascade may be a promising candidate for treatment of keloids, several hurdles must be addressed for such regimen to enter clinical evaluations; (1) transient therapeutic effect of Ad is not particularly well-suited for the treatment of chronically persistent keloids and (2) immunogenic nature of Ad vector could cause adverse inflammatory response. Further refinements to vector and treatment strategies will be required to achieve more efficient and safe gene transfer in clinical trials.

## Materials and Methods

### Human dermal fibroblast cell and keloid tissues

Keloid tissues were obtained from the central dermal layer of active-stage keloid patients (n = 11) (Supplementary Table 1) after acquiring informed consent, according to a protocol approved by the Yonsei University College of Medicine Institutional Review Board. HDFs and KFs were purchased from the American Type Culture Collection (ATCC, Manassas, VA). All experiments involving human tissues were performed in adherence to the Helsinki Guidelines. Separated cells were cultured in Dulbecco’s modified Eagle’s medium (DMEM; GIBCO, Grand Island, NY) supplemented with heat-inactivated 10% fetal bovine serum (FBS), penicillin (30 U/mL), streptomycin (300 μg/mL), and actinomycin (1 μg/mL).

### Adenoviral preparation and transduction efficiency in HDFs

The preparation of a replication-incompetent Ad with chimeric 5/35 knob expressing sLRP6E1E2 (dE1-k35/sLRP6E1E2) was described in a previous study^20^. The replication-incompetent Ad (dE1-k35/LacZ) was used as a negative control^11^. All viruses were propagated in human embryonic kidney 293 cells (HEK293; ATCC), purified by CsCl density purification, and then stored at −80 °C. Viral particle (VP) numbers were calculated from measurements of optical density at 260 nm (OD_260_) where 1 absorbency unit is equivalent to 10^12^ VP per milliliter. The MOI was calculated from VP.

To compare the transduction efficacy of control Ad (dE1/LacZ) and fiber-modified Ads (dE1-k35/LacZ and dE1-RGD/LacZ), we transduced HDFs with dE1/LacZ, dE1-k35/LacZ, or dE1-RGD/LacZ at either 5 or 10 MOI. β-galactosidase activity was visualized at 48 h post-transduction by 5-bromo-4-chloro-3-indolyl-β-D-galactopyranoside staining (Life Technologies, Gaithersburg, MD), as previously described^48^ (Supplementary Figure S5).

### Luciferase reporter assay for β-catenin activity

TOPFLASH luciferase reporter vectors (Upstate Biotechnology, Lake Placid, NY) were used to measure β-catenin/TCF signaling activity. A reporter plasmid (pTOPFLASH) contains three copies of optimal TCF-binding motif CCTTTGATC upstream of a minimal c-Fos promoter driving luciferase reporter gene^49^. HDFs and KFs were seeded (3 × 10^5^ cells) into 6-well plates and transfected with 0.3 µg of TOPFLASH (containing wild-type TCF binding sites) in conjunction with dE1-k35/LacZ or dE1-k35/sLRP6E1E2 (50 MOI) in serum-free DMEM. After 12 h, the medium was replaced with 1% DMEM with or without Wnt3a (100 ng/mL) or TGF-β1 (20 ng/mL), and then cells were incubated for another 24 hr. Cells were lysed with passive lysis buffer (Promega, Madison, WI), and 20 mL of the cell extract was analyzed by Dual-Luciferase Reporter Assay System (Promega). Experiments were carried out in triplicates and repeated at least three times.

### Quantitative real-time reverse transcriptase-polymerase chain reaction (qRT-PCR)

HDFs and KFs (5 × 10^5^ cells) were transduced with dE1-k35/sLRP6E1E2 or control Ad (dE1-k35/LacZ) in the presence of TGF-β1 (20 ng/mL) at 20, 50, and 100 MOI. At 3 days post-transduction, total RNA was prepared with TRIzol reagent (GIBCO), and complementary DNA was prepared from 0.5 μg of total RNA by random priming using a first-strand cDNA synthesis kit (Promega) as previously described^50^. Experiments were carried out in triplicates and repeated at least three times.

### Western blot analysis

To examine the effect of sLRP6E1E2 on the expression levels of LRP6, β-catenin, pGSK-3β, and Smad 2/3, HDFs were transduced with dE1-k35/LacZ or dE1-k35/sLRP6E1E2 in presence of Wnt3a (100 ng/mL) at 50 MOI. At 3 days after transduction, cells were lysed in a solution containing 50 mM Tris-HCl (pH 7.6), 1% Nonidet P-40 (NP-40), 150 mM NaCl, 0.1 mM zinc acetate, and protease inhibitors. Protein concentration was determined by the Lowry method (Bio-Rad, Hercules, CA), and 30 μg of each sample was separated by sodium dodecyl sulfate-polyacrylamide gel electrophoresis. The proteins from the gels were electrotransferred to polyvinylidene fluoride membrane and initially incubated with primary mouse anti-LRP6 (Santa Cruz biotechnology, Santa Cruz, CA), rabbit anti-β-catenin (Cell Signaling Technology, Beverly, MA), rabbit anti-pGSK-3β (Cell Signaling Technology), rabbit anti-Smad 2/3 (Cell Signaling Technology), or rabbit anti-β-actin antibody (Sigma, St Louis, MO), and then secondarily incubated with the horseradish peroxidase-conjugated secondary antibody (Cell Signaling Technology). The expression patterns were revealed using enhanced chemiluminescence detection kit (Santa Cruz Biotechnology). And the expression levels of LRP6, β-catenin, pGSK-3β, and Smad 2/3 were semi-quantitatively analyzed using ImageJ software (National Institutes of Health, Bethesda, MD).

### Enzyme-linked immunosorbent assay (ELISA) for TGF-ß1 expression

HDFs and KFs (5 × 10^5^ cells) in 6-cm culture dishes were transduced with dEl-k35/LacZ or dEl-k35/sLRP6E1E2 at 50 MOI. At 3 days post-infection, the supernatant was collected by centrifugation at 15,000 *g* for 10 min at 4 °C, and the level of TGF-β1 expression was assessed using an ELISA kit (R&D Systems, Minneapolis, MN) according to the manufacturer’s recommendations.

### Immunofluorescence assay

For immunofluorescence microscopy, cultured cells were washed twice with phosphate buffered saline (PBS), fixed in 4% paraformaldehyde for 10 min at room temperature, and then permeabilized by incubation for 15 min with 0.1% Triton X-100 in PBS. The samples were blocked with 1% bovine serum albumin followed by incubation with rabbit anti-Smad 2/3 or rabbit anti-β-catenin (Abcam, Ltd., Cambridge, UK) primary antibody overnight at 4 °C. One day after incubation, cells were washed with PBS and incubated with Alexa Flour 488-conjugated goat anti-rabbit IgG (invitrogen, Carlsbad, CA) or Alexa Flour 633-conjugated goat anti-rabbit IgG (invitrogen) secondary antibody, respectively, for 60 min at room temperature. The final antibody treatment also contained tetramethylrhodamine isothiocyanate-conjugated phalloidin (Sigma) for actin and DAPI for nucleus staining (both at 1 µg/mL, Sigma). The slides were mounted with Vectashield mounting medium (Vector Laboratories, Burlingame, CA) and cells were viewed under a confocal laser-scanning microscope (LSM510, Carl Zeiss MicroImaging, Thornwood, NY).

### Primary keloid tissue explants culture

Keloid tissues were obtained from active-stage keloid patients (n = 4). Keloid tissue explants were prepared by dissecting keloid central dermal tissue into 2-mm diameter pieces with sterile 21-gauge needles. The explants were plated onto HydroCell^®^ 24 multi-well plates (Nunc, Rochester, NY) after which they were cultured for 4 hr in Isocove’s modified Dulbecco’s medium (GIBCO) supplemented with 5% FBS, 10 mM insulin (Sigma) and 1 mM hydrocortisone (Sigma). For histological analysis, dEl-k35/LacZ or dEl-k35/sLRP6E1E2 at 1 × 10^10^ VP or 10 mM of commercially available Wnt inhibitor, JW 55 (Axon Medchem, Groningen, Netherland), were added into the plates containing keloid tissue explants, and incubated at 37 °C in 5% CO_2_ incubator for 3 or 5 days.

### Histology and immunohistochemistry

The transduced keloid tissue explants were then fixed with 10% formalin, paraffin-embedded, and cut into 5-μm-thick sections. Representative sections were stained with Masson’s trichrome or Picrosirius red, and then examined by light microscopy. For immunohistochemical staining, keloid tissue explants sections were incubated at 4 °C overnight with mouse anti-Wnt3a (Abcam), rabbit anti-TGF-β1 (Abcam), mouse anti-collagen type-I (Abcam), mouse anti-collagen type-III (Sigma), mouse anti-elastin (Sigma), mouse anti-fibronectin (Santa Cruz Biotechnology), rabbit anti-MMP-9 (Abcam) primary antibody, and then incubated at room temperature for 20 min with the Dako Envision™ Kit (Dako, Glostrup, Denmark) as a secondary antibody. Diaminobenzidine/hydrogen peroxidase (Dako) was used as the chromogen substrate. All slides were counterstained with Meyer’s hematoxylin. The expression levels of Wnt3a, TGF-β1, collagen type-I, collagen type-III, elastin, and fibronectin were semi-quantitatively analyzed using MetaMorph^®^ image analysis software (Universal Image Corp., Buckinghamshire, UK). Results are expressed as the mean optical density of six different digital images.

### Statistics analysis

Results were expressed as the mean ± standard deviation (SD). Data were analyzed by one-way ANOVA for assays comparing more 3 experimental groups or more and a paired t-test for assays comparing two experimental groups. *P* < *0.05* was considered statistically significant.

## Electronic supplementary material

Supplementary information
