# Supplementary material for: Decoy Wnt receptor (sLRP6E1E2)-expressing adenovirus induces anti-fibrotic effect via inhibition of Wnt and TGF-β signaling
Source: Sci Rep. 2017 Nov 8;7:15070. doi: 10.1038/s41598-017-14893-w (PMC5678438; doi:10.1038/s41598-017-14893-w)

Decoy Wnt receptor (sLRP6E1E2)-expressing adenovirus induces anti-fibrotic effect via inhibition of Wnt and TGF-β signaling

Won Jai Lee[1], Jung-Sun Lee[2], Hyo Min Ahn[2], Youjin Na[2], Chae Eun Yang[1], Ju Hee Lee[3], JinWoo Hong[2], Chae-Ok Yun[2*]

[1]Institute for Human Tissue Restoration, Department of Plastic & Reconstructive Surgery, Yonsei University College of Medicine, Seoul, Korea, [2]Department of Bioengineering, College of Engineering, Hanyang University, 222 Wangsimni-ro, Seongdong-gu, Seoul 133-791, Korea, and [3]Department of Dermatology, Yonsei University College of Medicine, Seoul, Korea

Supplementary Table 1. Demographic information and descriptions of the keloids from the patients who participated in this study.

Case (No.)	Sex	Age (years)	Keloid Site	Others
K1	M	31	Shoulder	IHC, qPCR
K2	F	50	Chest	IHC
K3	F	17	Ear lobe	IHC
K4	F	44	Ear lobe	IHC, qPCR
K5	M	53	Shoulder	IHC, qPCR
K6	F	33	Earlobe	Tissue explant culture, IHC
K7	F	18	Ankle	Tissue explant culture, IHC
K9	M	28	Neck	Tissue explant culture, IHC
K10	F	72	Earlobe	Western blot
K11	F	32	Earlobe	Tissue explant culture, IHC

IHC, immunohistochemistry; qPCR, quantitative polymerase chain reaction.

Supplementary Figure Legends

Supplementary Figure S1. Histological and immunohistochemical analysis for Wnt3a and β-catenin expression in human keloid tissues. (a) Keloid tissue (Patients K1) displayed dense and excessive collagen deposition that extends over clinical keloid margin into the normal tissue. Higher levels of both Wnt3a and β-catenin expression were observed in the region of the keloid tissues than those in adjacent normal tissue. Original magnification: ×100 or ×400. Red dotted line: boundary between keloid and normal region (b, c) Semi-quantitative analysis by MetaMorph® image analysis software. The data are representatives of three independent experiments (**p < 0.01).

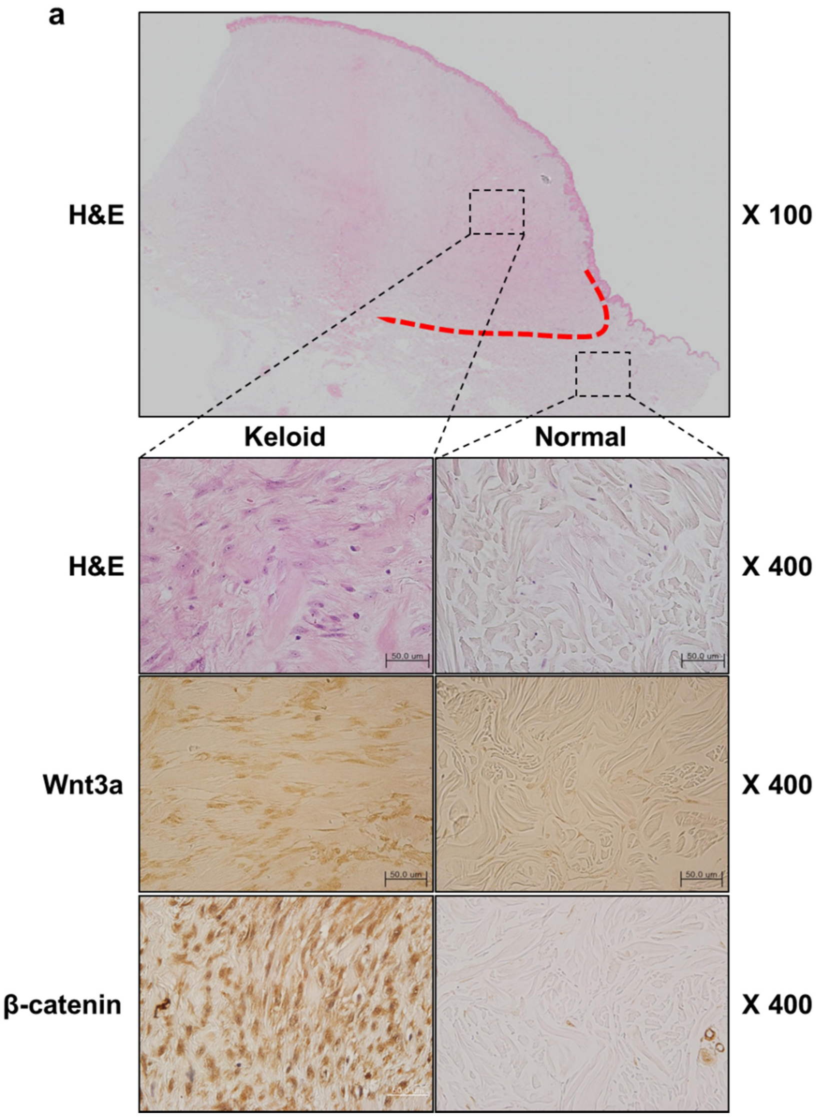

b. Wnt3a

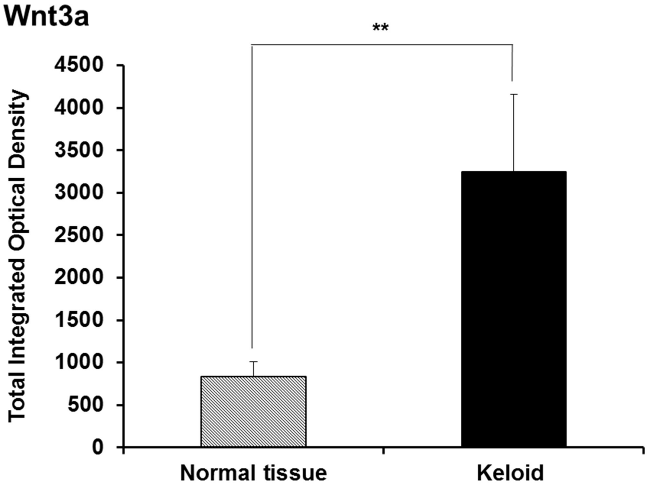

c. β-catenin

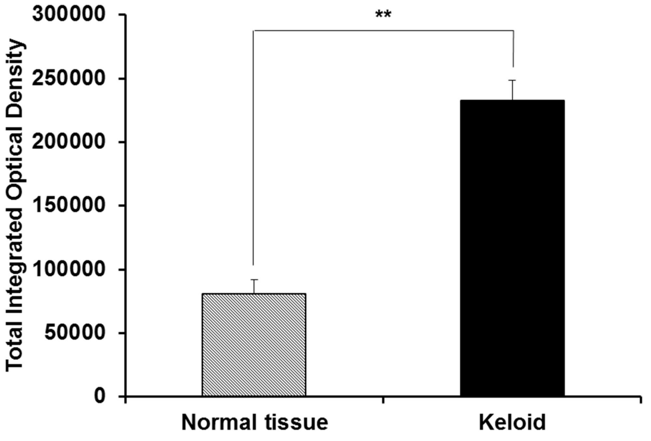

Supplementary Figure S2. The decreased expression of Wnt3a (a) and TGF-β1 (b) was significantly observed in keloid tissue explants (Patients K6, K7, K9) treated with dEl-k35/sLRP6E1E2 in comparison to those treated with dE1-k35/LacZ.

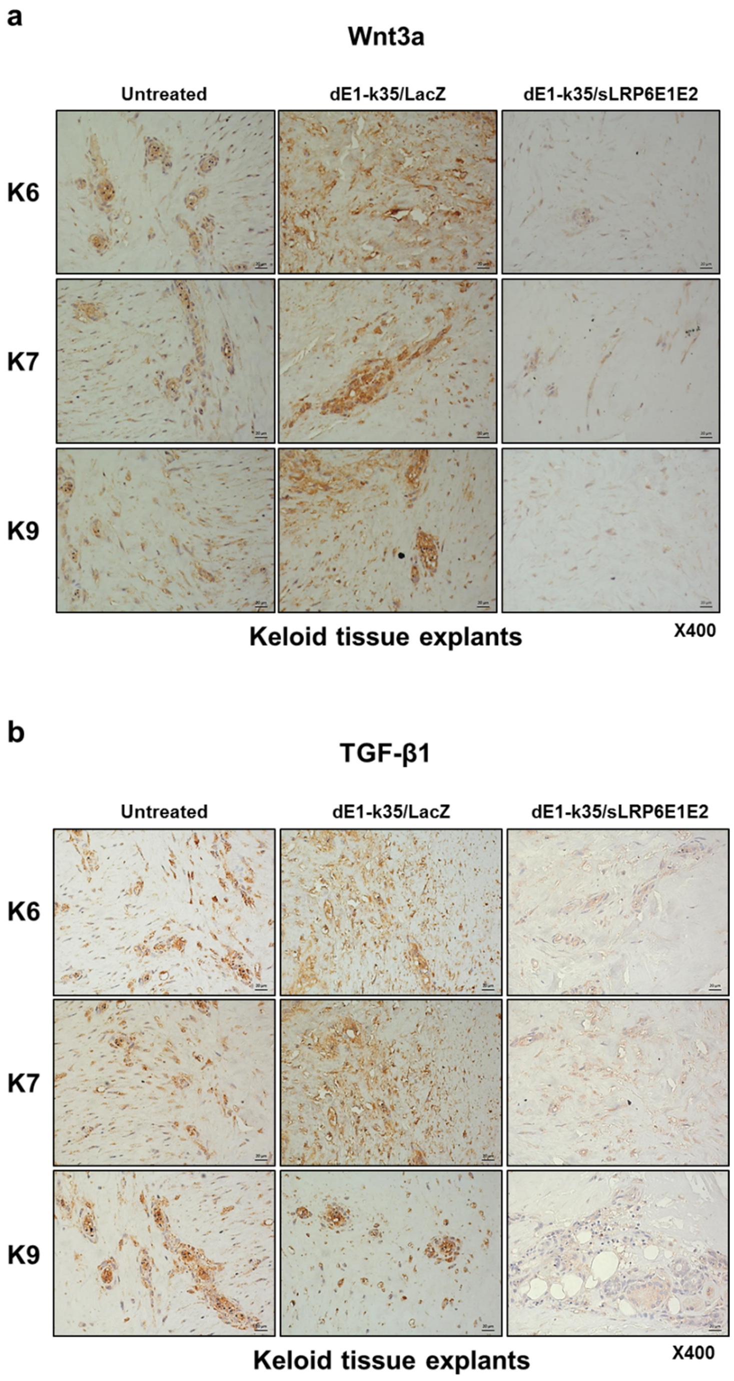

Supplementary Figure S3. A decrease of type-I and -III collagen protein levels was confirmed by western blot analysis in dEl-k35/sLRP6E1E2-treated keloid tissue explants (Patients K10).

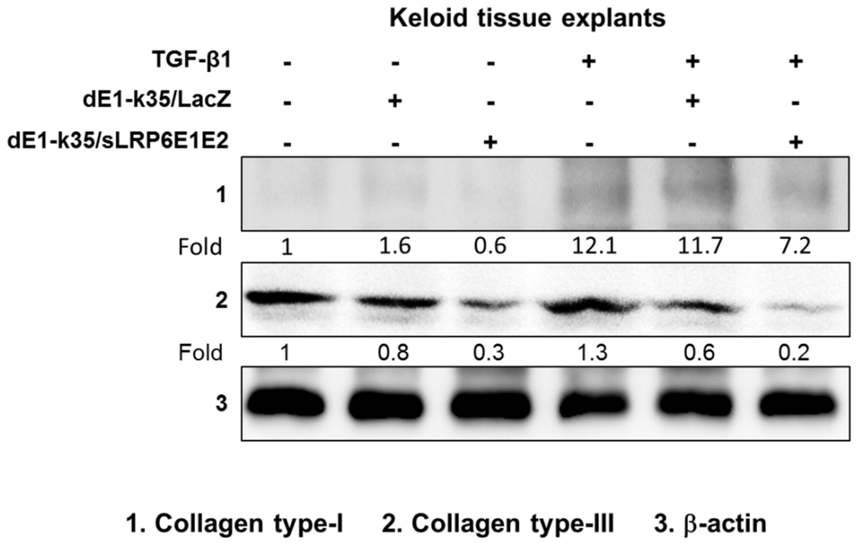

Supplementary Figure S4. The expression levels of matrix metalloproteinase (MMP)-9 in keloid tissue explants (Patients K6, K7, K9) transduced with dEl-k35/sLRP6E1E2 was reduced by 15% and 4%, respectively, in comparison to dE1-k35/LacZ-treated tissue explants.

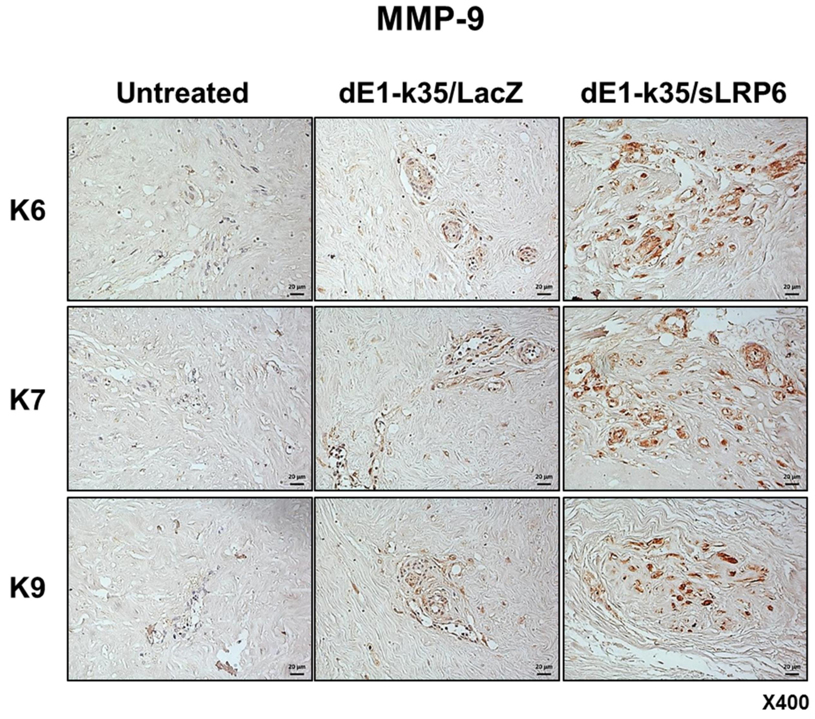

Supplementary Figure S5. 5-bromo-4-chloro-3-indolyl-β-D-galactopyranoside staining for β-galactosidase activity in HDFs after transduction with dE1/lacZ, dE1-k35/lacZ, or dE1-RGD/lacZ (5 or 10 MOI). HDFs demonstrated higher susceptibility to Ad with k35 fiber modification (dE1-k35/lacZ) than Ads containing either wild-type (dE1/lacZ) or RGD-modified (dE1-RGD/lacZ) fibers.

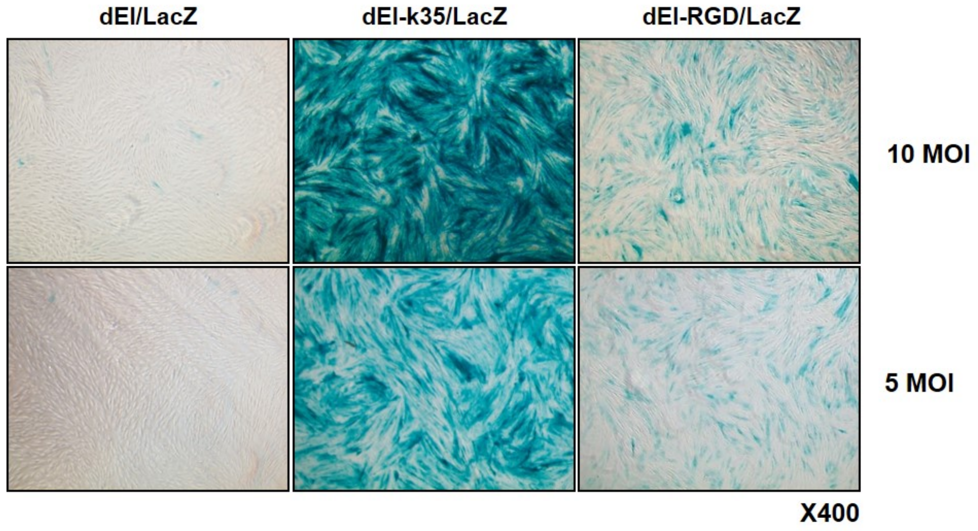

Supplement: Supplementary file 1 — Supplementary information [file 41598_2017_14893_MOESM1_ESM.pdf]
